# Maize Global Transcriptomics Reveals Pervasive Leaf Diurnal Rhythms but Rhythms in Developing Ears Are Largely Limited to the Core Oscillator

**DOI:** 10.1371/journal.pone.0012887

**Published:** 2010-09-23

**Authors:** Kevin R. Hayes, Mary Beatty, Xin Meng, Carl R. Simmons, Jeffrey E. Habben, Olga N. Danilevskaya

**Affiliations:** Pioneer Hi-Bred International, a DuPont Company, Johnston, Iowa, United States of America; University of Massachusetts Amherst, United States of America

## Abstract

**Background:**

Plant diurnal rhythms are vital environmental adaptations to coordinate internal physiological responses to alternating day-night cycles. A comprehensive view of diurnal biology has been lacking for maize (*Zea mays*), a major world crop.

**Methodology:**

A photosynthetic tissue, the leaf, and a non-photosynthetic tissue, the developing ear, were sampled under natural field conditions. Genome-wide transcript profiling was conducted on a high-density 105 K Agilent microarray to investigate diurnal rhythms.

**Conclusions:**

In both leaves and ears, the core oscillators were intact and diurnally cycling. Maize core oscillator genes are found to be largely conserved with their Arabidopsis counterparts. Diurnal gene regulation occurs in leaves, with some 23% of expressed transcripts exhibiting a diurnal cycling pattern. These transcripts can be assigned to over 1700 gene ontology functional terms, underscoring the pervasive impact of diurnal rhythms on plant biology. Considering the peak expression time for each diurnally regulated gene, and its corresponding functional assignment, most gene functions display temporal enrichment in the day, often with distinct patterns, such as dawn or midday preferred, indicating that there is a staged procession of biological events undulating with the diurnal cycle. Notably, many gene functions display a bimodal enrichment flanking the midday photosynthetic maximum, with an initial peak in mid-morning followed by another peak during the afternoon/evening. In contrast to leaves, in developing ears as few as 47 gene transcripts are diurnally regulated, and this set of transcripts includes primarily the core oscillators. In developing ears, which are largely shielded from light, the core oscillator therefore is intact with little outward effect on transcription.

## Introduction

The day-night cycle is a major environmental cue that controls both daily and seasonal rhythms in plants. Diurnal light-dark transitions entrain the internal circadian clock that generates rhythms which are self-sustained (free-running) under constant light conditions. A simplified model of the clock comprises three basic components: an input pathway that senses light, a core oscillator that is the transcriptional machinery defining the rhythms, and output pathways that control various developmental and metabolic processes, resulting in the appropriate physiological adaptations to the day-night cycle (reviewed [1], [2]). The proper synchronization of the internal clock and external light/dark cycles results in better plant fitness, survival, competitive advantage [3] and growth vigor [4].

To date, the genetic architecture of the plant circadian system has been elucidated largely in *Arabidopsis thaliana* [5], [6]. The input pathways comprise two sets of photoreceptors, the red/far-red sensing phytochromes (PHYA-E) and the UV-A/blue-light sensing cryptochromes (CRY1 and CRY2), which perceive light during the day and send signals to the core oscillator [7]. In turn, the core oscillator genes form interlocking transcriptional feedback loops [2], [7]. The morning loop consists of the MYB-like transcription factors *CCA1 (CIRCADIAN CLOCK ASSOCIATED 1)* and *LHY (LATE ELONGATED HYPOCOTYL)*, which participate in the regulation of two different loops. In the morning loop, CCA1/LHY negatively regulate transcription of the pseudo-response regulator *TOC1 (TIMING OF CAB EXPRESSION 1)* and the TCP-like transcription factor *CHE (CCA1 HIKING EXPEDITION)* [8]. TOC1 is a positive transcriptional regulator of CCA1/LHY through partial inhibition of *CHE* repression of *CCA1*. In the day loop, CCA1/LHY positively regulate transcription of *PRR5*,*PRR7* and *PRR9 (PSEUDO-RESPONSE REGULATORS*), both of which negatively regulate *CCA1/LHY* [9]. In the evening loop, TOC1 through CCA1/LHY works as a negative regulator of *GI (GIGANTEA)*, itself a positive regulator of *TOC1*. The evening gene *ZTL* (*ZEITLUPE*, a protein-degrading F-box protein), involved in degradation of TOC1 and PRR3 proteins, provides regulation of the core clock components at the protein level [10]. The multiple interlocking transcription loops maintain a robust yet flexible regulatory machinery [2].

The circadian clock generates rhythmic outputs that regulate many plant developmental and physiological processes including: growth [11], [12], flowering time, tuberization in annuals, growth cessation and bud set in perennials [13], photosynthesis [14], nitrogen uptake [15], as well as hormone signaling and stress response [16]. Specific links of the circadian clock with output pathways are still being discovered. So far the best understood connection is the photoperiod regulation of flowering time in Arabidopsis and rice. The Arabidopsis clock gene *GI* and its rice ortholog *OsGI* promote expression of the transcription factors *CO (CONSTANS)* and *OsCO (Hd1*, *HEADING DATE 1)*, which control transcription of the downstream floral activator *FT (FLOWERING LOCUS T)* in Arabidopsis and its homologous gene *Hd3a (HEADING DATE 3a)* in rice [17], [18]. These photoperiod sensitive pathways ensure flowering under favorable conditions.

Several recent publications identified molecular connections between the Arabidopsis core oscillators and a broad range of plant physiological processes. Rhythmic hypocotyl growth is promoted by positive action of two basic helix-loop-helix transcription factors, *PIF4* and *PIF5* (*PHYTOCHROME-INTERACTING FACTOR)* whose transcript levels are regulated by *CCA1* [11]. Hypocotyl growth is also independently regulated by the phytohormone auxin, produced by the auxin biosynthetic gene *YUCCA8*, that is controlled directly by the clock-dependent Myb-like transcription factor RVE1 (REVEILLE 1) [19]. This is a direct link between circadian oscillators and the auxin networks that coordinate seedling growth in Arabidopsis. Output pathways of *PRR9/7/5* genes are related to maintenance of central metabolism, mainly in mitochondria, and in particular the tricarboxylic acid (TCA) cycle [20]. *TOC1* is also linked with the stress-related abscisic acid (ABA) hormone connecting circadian clocks with plant responses to drought [21].

Diurnal rhythms under day/night cycles are generated by environmental cues such as light and temperature, and by internal cues such as the free-running circadian clock and sugar availability [22], [23]. The use of microarray technology has uncovered the pervasive diurnal rhythms in protein-encoding transcriptomes of light-sensing tissues for the model plant Arabidopsis [24], [25] and crop plants such as tomato [26] and soybean [27]. Depending on the experimental conditions and tissue studied, the estimated number of diurnally expressed genes may vary from 11% in Arabidopsis rosette leaves [25] and 15% in tomato seedlings [26] and up to 89% also in Arabidopsis rosette leaves [23]. Depending on the computational algorithms employed, the oscillation pattern could be revealed for almost every gene in Arabidopsis [28]. Moreover, a whole Arabidopsis genome tilling array uncovered the circadian regulation of the non-coding transcriptome, including micro- and small-RNAs, and up to 7% of antisense transcripts [29]. Many gene functional categories such as metabolic and signaling pathways are affected by diurnal environmental stimuli such as light and temperature, revealing a complex physiological system, often with overlapping interactions with systems such as response to sugar availability [22], [23], [30].

No systematic study of diurnal/circadian transcriptional patterns in maize has been reported. In the pre-genomic era, diurnal changes were observed in maize leaf photosynthesis and in leaf elongation rates, which reached their maximum activity at midday [31], [32]. Diurnal oscillation of the endosperm-specific transcription factor O2 *(Opaque 2)* was also found in non-photosynthetic kernels, and it was proposed that O2 transcription might be controlled by diurnal metabolite flux [33]. Circadian regulation of the maize *Cat3* catalase gene was reported [34]. Diurnal and circadian rhythms were demonstrated for maize homologues of *GI* (gigz1A, B) and *CO (conz1)*, which are direct outputs of the circadian clock in the photoperiod pathway controlling Arabidopsis flowering time [35], even though temperate maize is a day-neutral plant whose flowering is not sensitive to photoperiod.

The present study was initiated to examine the extent to which the diurnal cycle regulates gene transcription in maize using genome-wide profiling. A field experiment was designed under natural conditions where a photosynthetic tissue, the leaf, and a non-photosynthetic tissue, the developing ear, were sampled at four-hour intervals for three consecutive days. In this report a high-density Agilent microarray was used to identify thousands of transcripts that cycle diurnally in maize leaves. These leaf cycling transcripts share many characteristics with previously identified diurnally responsive genes in Arabidopsis. In contrast, non-photosynthetic developing ears exhibited only a small number of genes with clear diurnal cycling. Many of these are maize homologues of Arabidopsis core oscillator genes, suggesting that core circadian genes are conserved in maize and diurnally expressed in both photosynthetic and non-photosynthetic tissues, but with an attenuated response in the latter.

## Results

### Diurnal Rhythms Measured

Maize plants (B73 inbred line) were grown under replicated field conditions and sampled at V14-15 stage (Materials and Methods). Light conditions at sampling were approximately 14.75 hours of sunlight according to records of U.S. Naval Observatory. Starting at sunrise on day 1, the topmost fully expanded leaves and primary ears were sampled at 4 hour time intervals over three consecutive days. As sunrise on the first day was 6:01AM, we hereafter use 6AM as ZT0 (Zeitgeber time). This sampling strategy was used effectively in other studies to elucidate circadian rhythms [36], [37]. Messenger RNA profiling was performed on custom Agilent maize arrays designed to interrogate global gene expression patterns across circa 105 K probes (Materials and Methods).

The GeneTS methodology was applied to the data to determine periodicity [38]. This method first creates a periodogram for Fourier frequencies. Significant Fourier frequencies are then assessed for significance via Fisher's g-statistic. Given the experimental design, this method shows greater power to detect circadian rhythmicity than other commonly used methods [39], [40]. The significance values from Fisher's g-Test were then corrected for multiple measures comparisons via conversion to q-values to assess False Discovery Rates (FDR) [41]. Diurnally regulated transcripts were determined as those having significant expression at least once per day and also that were significant at q<0.001.

### Diurnal Agilent Microarray Analysis

**Figure 1:**
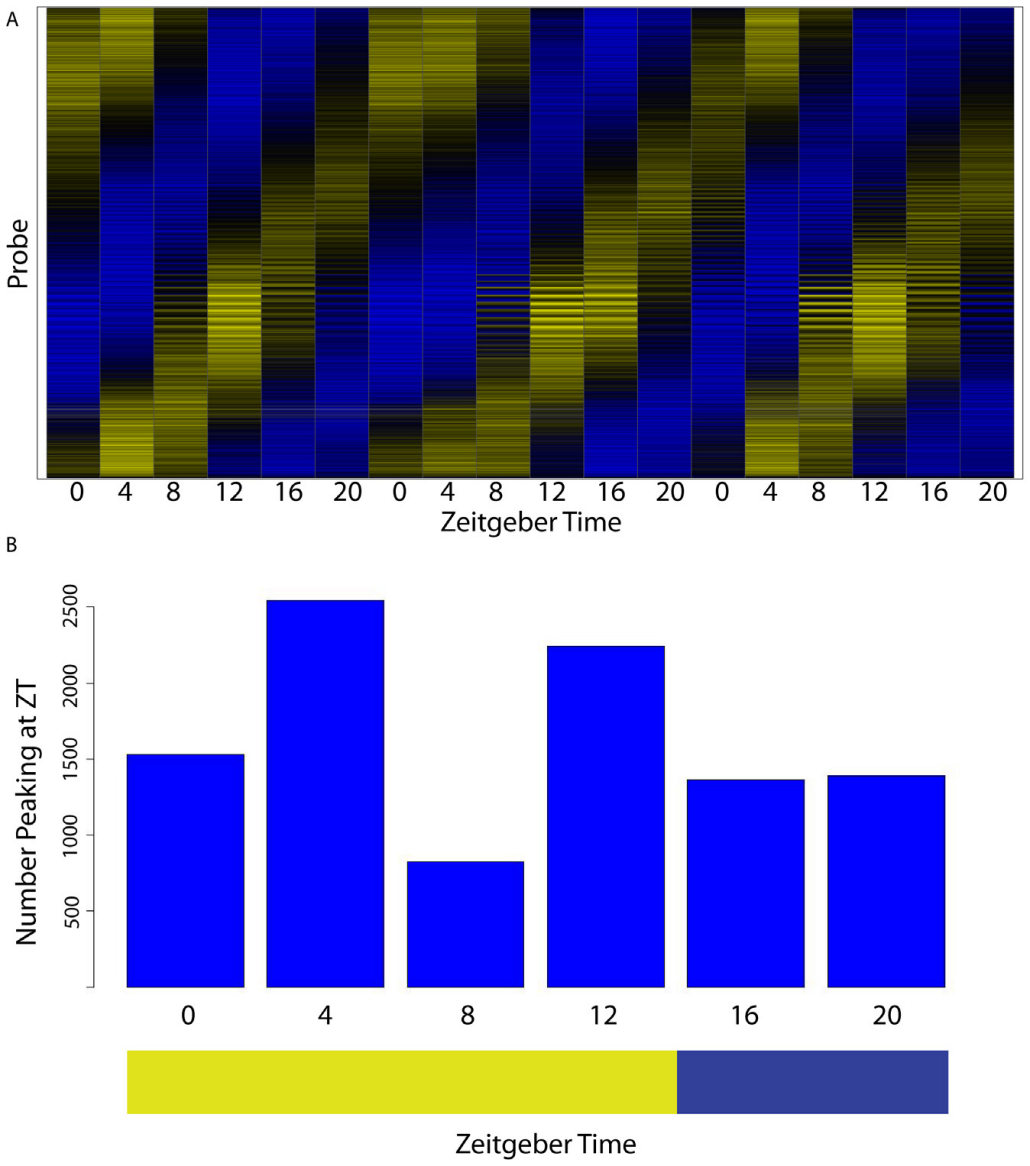
Gene expression in maize leaf. As assayed by Agilent microarray analysis, 10,037 transcripts were identified as diurnally cycling with a period near 24 hours via the GeneTS method. A) A heat map showing normalized gene expression patterns of significant transcripts. Each transcript is normalized to the median of that transcript's signal intensity. Color scale runs from 0.25 (blue) to 4 (yellow). B) Phase diagram of significant cycling transcripts, as determined by greatest mean expression. Time points immediately following light/dark transitions are enriched for peak expression.

Diurnal rhythms of gene expression were readily detectable within the photosynthetic leaf tissue (Figure 1A). Of the 44,187 probes with detectable expression, 10,037, or 22.7%, were identified as cycling by the GeneTS algorithm (Table 1). A complete set of probes cycling in leaf tissues is shown in Table S1. While this analysis likely reveals a conservative estimate of the cycling patterns, the proportion of cycling transcripts here is consistent with the proportion reported for Arabidopsis [24], [25], [29]. Amplitudes of cycling transcripts are robust, with a median peak/trough ratio circa 5-fold, with many showing peak/trough ratios of higher than 20-fold. The peak expression for these cycling transcripts exhibits a broad distribution across all phases of the day (Figure 1B).

**Table 1:** Summary of diurnal rhythms by tissue.

Assay	# Time Points	#Samples	Probes Detected	Probes Cycling	Percentage Cycling
Agilent Leaf	18	54	44,187	10,037	22.71%
Agilent Ear	18	54	38,445	149	0.39%

**Figure 2:**
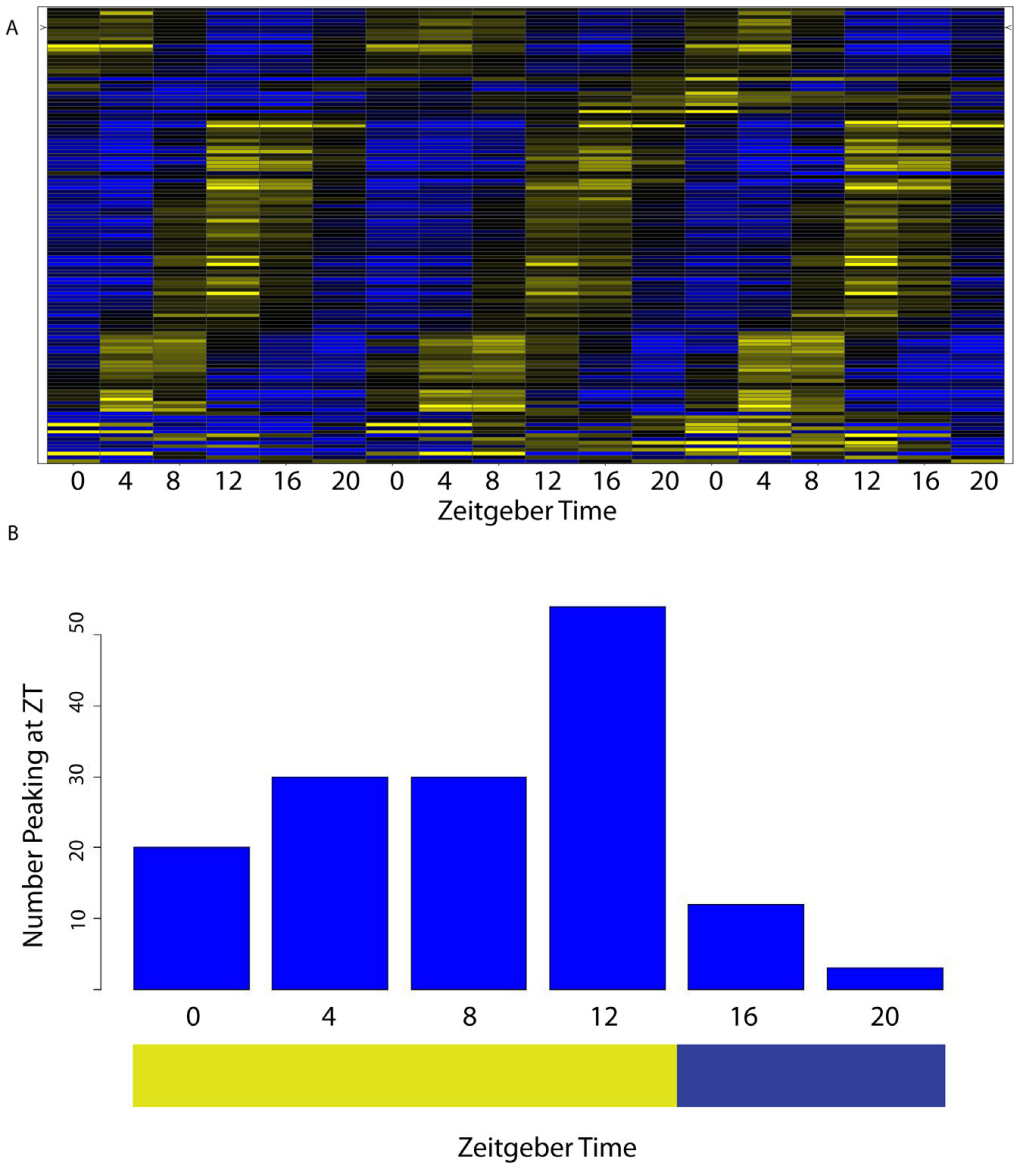
Gene expression in maize developing ears. As assayed by Agilent microarray analysis, 149 transcripts were identified as cycling with a period near 24 hours via the GeneTS method. A) A heat map showing normalized gene expression patterns of significant transcripts. Each transcript is normalized to the median of that transcript's signal intensity. Color scale runs from 0.25 (blue) to 4 (yellow). B) Phase diagram of significant cycling transcripts, as determined by greatest mean expression. Despite low numbers of cycling transcripts, there appears to be enrichment for early evening peak expression.

In contrast to the leaf results, very few probes within the developing ear exhibited diurnal rhythms (Figure 2A). Only 149 of the 38,445 expressed transcript probes (0.39%) were positively identified as cycling (Table 1, Figure S1), an approximately 96% reduction in the number of detected cycling probes relative to that of leaves. Despite the low number of cycling probes, there is early-evening enrichment, with roughly one-third of the cycling transcripts peaking in this phase (Figure 2B). Of these 149 cycling probes, 100 of them (67.1%) were also diurnally cycling in the leaf tissue. Among those that cycled in both leaf and ear tissues, amplitudes of the rhythms are severely attenuated in the developing ear (Table S2). This list was subsequently reduced to 47 putative ear cycling genes after consolidation of redundant probes and thorough gene annotation (Table S3). Many of these genes appeared to be maize homologs of Arabidopsis core clock genes *CCA1/LHY, TOC1, PRR7/3*, *GI*, and *FKF1* (flavin binding, kelch repeat, F box protein). The maize ear tissue core oscillator thus appears to be intact, but is apparently decoupled from the majority of its transcriptional output systems.

### Core Clock Components

A host of core components of the Arabidopsis circadian oscillator has been identified [2]. In order to assess whether these components also play a role in maize rhythmic expression, the best maize orthologs were identified by reciprocal BLASTp searches. Putative orthologs were further evaluated to determine whether the inferred protein relationships abide by the speciation pattern, and were then queried for diurnal oscillation patterns in leaf and ear tissues on the Agilent microarray. Employing these criteria, maize orthologs were identified for several major core components including *CCA1/LHY*, *TOC1*, *PRR7/3/9*, *GI*, and *FKF1* (Table 2). The maize *CHE* ortholog, the integrator of *TOC1* function [8], was identified on chromosome 5 (BAC ID AC209463). This gene encodes a protein with 47% identity and 53% similarity to Arabidopsis CHE; however, on the Agilent microarray the maize *CHE* mRNA does not display a diurnal pattern.

**Table 2:** Maize core clock genes.

Candidate gene^a^	Annotation	*Zm* Phase	*At* Phase^b^	*Os* Phase^c^	Probes ID	BAC ID	*Zm* Chr	GenBank cDNA
*ZmCCA1*	myb transcription factor	ZT4	ZT1	ZT1	A4204614	AC196205	4	NM_001154010
*ZmLHY*	myb transcription factor	ZT4	ZT1	ZT1	A4246692	AC213378	10	NM_001138057
*ZmTOC1a*	pseudo-response regulator	ZT12	ZT12	ZT12	A4271366	AC195353	4	NM_001154351
*ZmTOC1b*	pseudo-response regulator	ZT12			A4244614	AC196675	5	HQ003892
*ZmPRR73*	pseudo-response regulator	ZT4	ZT8	ZT4	A4313747	AC205568	9	EU952116
*ZmPRR37*	pseudo-response regulator	ZT4	ZT11	ZT6	A4303803	AC195801	7	EU952111
*ZmPRR59*	pseudo-response regulator	ZT12	ZT7	ZT12	A4241676	AC210923	10	HQ003893
*ZmGI1A*	gigantea *(gigz1A)*	ZT12	ZT8		A4249658	AC193462	8	BK006299
*ZmGI1B*	gigantea *(gigz1B)*	ZT12			A4229306	AC202834	3	BT039477
*ZmFKF1a*	flavin binding, kelch repeat, F box protein	ZT12	ZT8	ZT8	A4260413	AC190896	4	NM_001152685
*ZmFKF1b*	flavin binding, kelch repeat, F box protein	ZT12			A4289207	AC203828	2	EU954587

a Two maize (*Zm*) candidates were found for a single Arabidopsis gene *TOC1*, *GI* and *FKF1*

b The phases of Arabidopsis (*At*) clock components were defined by using the on line tool “DIURNAL” http://diurnal.cgrb.oregonstate.edu/index.html under the diurnal long day conditions which are the most similar conditions to our maize field experiment.

c The phase of rice (*Os*) clock components were based on published data [43], [48].

In some cases we were unable to sufficiently discriminate between candidate orthologous proteins at the amino acid similarity level and so we named them arbitrarily. For example, maize proteins encoded by *ZmCCA1* (chromosome 4) and *ZmLHY* (chromosome 10) do not have an unambiguous one-to-one homology with Arabidopsis proteins. The maize gene models for *ZmCCA1* and *ZmLHY* are encoded by long genic regions of circa 45 kb and 73 kb, respectively (Figure S2). Maize genes of this size are extremely rare, where the average gene size is 4 kb [42].

Two *TOC1* homologs, *ZmTOC1a* and *ZmTOC1b*, were found and mapped to chromosome 4 and 5, respectively (Table 2). Transcription of both genes peaks at ZT12, consistent with Arabidopsis *TOC1* gene expression patterns. *TOC1* is a member of the pseudo-response regulator (PRR) family composed of five evolutionarily conserved *PRR* genes in Arabidopsis and rice [43], [44]. In addition to two *ZmTOC1* homologs, we identified *ZmPRR73*, *ZmPRR37* and *ZmPRR59* that were named after rice *PRR* genes based on the level of protein similarly [43].

Four maize *ZEITLUPE* homologs [45] were indentified by protein homology. They shared conserved domains with Arabidopsis proteins FKF1 (flavin binding, kelch repeat, F box protein) [46] and LKP2 (LOV kelch protein2) [47] (Figure S3). *ZmFKF1a* and *ZmFKF1b* are cycling in both tissues and peaking in the evening, which make them candidates for the Arabidopsis *FKF1* gene. They are mapped to chromosome 4 and 2, respectively. It is worth noting that *ZmFKF1b* contains only one kelch repeat and is thus diverged from the consensus. *ZTL* does not appear to cycle at the mRNA level, but rather the protein level in Arabidopsis and rice [45], [48]. It is therefore expected that the maize *ZTL* ortholog would have the similar mode of regulation at the protein level.

**Figure 3:**
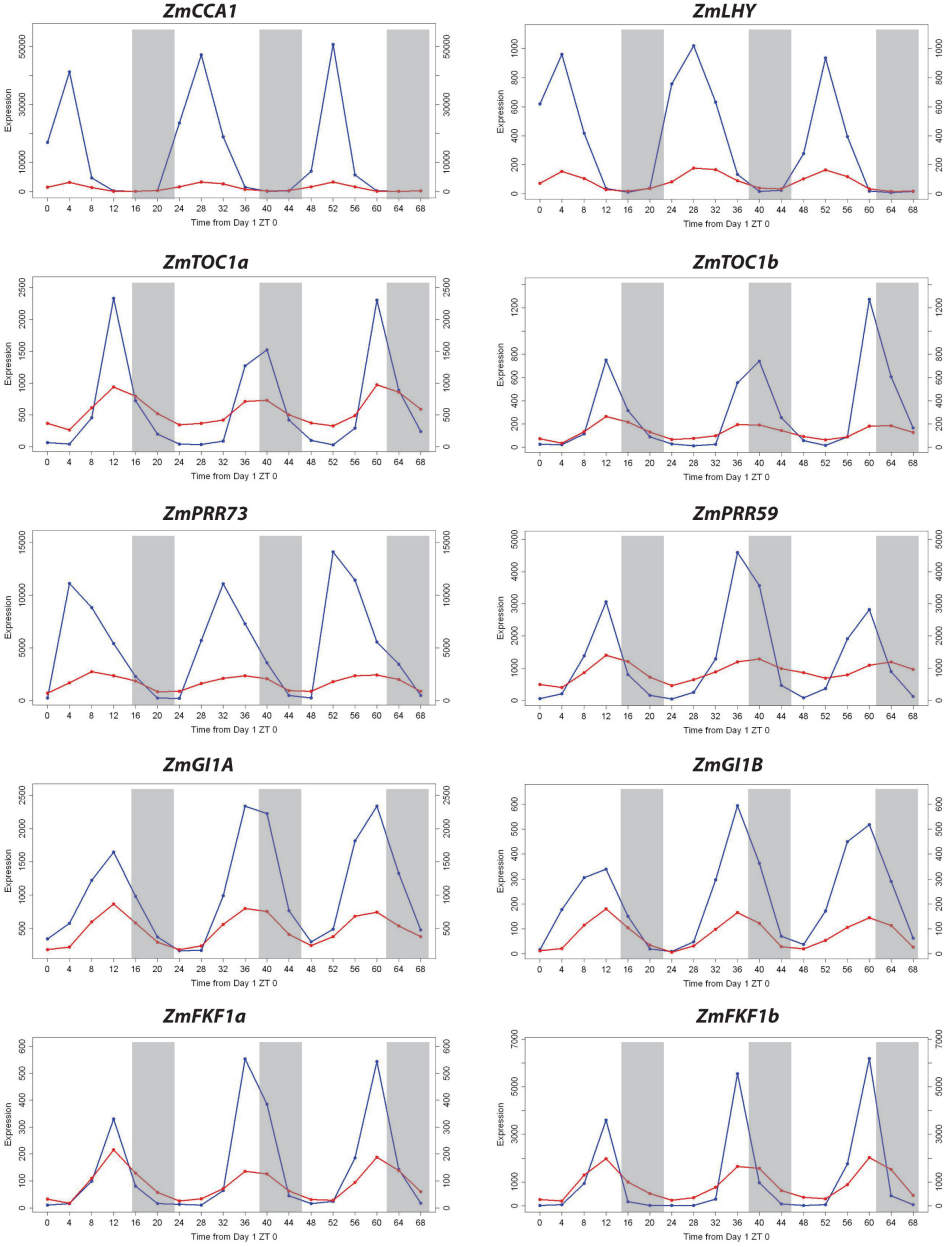
Diurnal oscillation of the core clock components. Leaf transcripts are plotted in blue, and ear data are plotted in red, overlaid with light/dark cycles for: *ZmCCA1*, *ZmLHY*, *ZmTOC1a*, *ZmTOC1b*, *ZmPRR73*, *ZmPRR59*, *ZmGI1A (gigz1A)*, *ZmGI1B (gigz1B)*, *ZmFKF1a* and *ZmFKF1b*.

Two maize orthologs of *GIGANTEA*, *gigz1A* and *gigz1B*, were described previously [35], and their diurnal oscillation is confirmed in both ears and leaves in our experiment. Identified maize core components were shown to cycle in both leaves and developing ears and the phases are conserved between plant species [48] (Table 2 and Figure 3). Cycling of the core components *ZmCCA*, *ZmLHY*, *ZmTOC1a* and *ZmTOC1b* was further confirmed via qRT-PCR analysis (Figure S4). The amplitudes of the core components are attenuated in developing ears when compared with leaf tissue, but still robust. These data show that most of the maize clock homologs are diurnally expressed in non-photosynthetic tissues such as the ear, but the oscillator output is largely isolated from the transcriptional machinery affecting downstream diurnal expression changes.

### Leaf Diurnal Output Pathways

**Figure 4:**
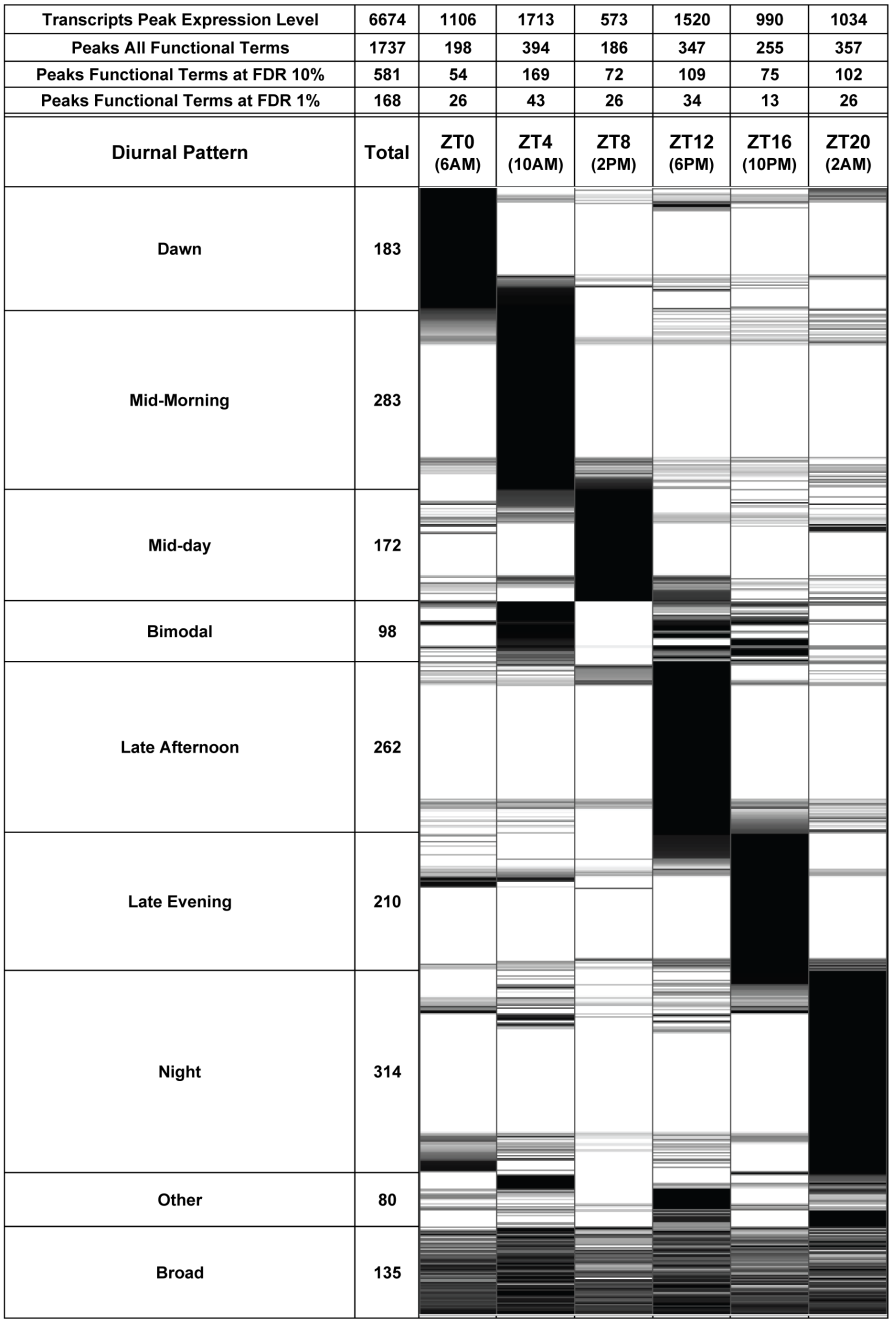
Diurnal Representation Patterns of 1737 Gene Functional Terms. 1737 Gene Functional Terms were horizontally categorized by the time point at which their representation peaks, and vertically into descriptive diurnal patterns, such as ‘Dawn’ or ‘Bimodal’. The gray-scale plot is the percent of maximum FEI for the six time points. FEI stands for Functional Enrichment Index, which is the absolute Log of the binomial probability (see Material and Methods). The table at the top shows the number of transcripts and functional terms peaking at the six time points, and also the number of functional terms that peaked at those time points following filtering at a 10% and 1% FDR (false discovery rate).

This study reveals that diurnally regulated transcripts pervade most functions of the maize leaf cells. We ascribed the 10,037 diurnally-regulated Agilent probes to 6674 transcripts, which represent over 22% of the total detected transcripts expressed, and these transcripts could be assigned to a diverse set of 1737 different Gene Ontology (GO) EuKaryotic Orthologous Groups (KOG) functional categories (Figure 4 and Table S4). Considering that this diurnal study almost certainly underestimates the total number of diurnally regulated transcripts, owing to factors such as the stringency of the diurnal pattern filter cutoff, the resolution limitations of just six 4-hour time points, technical vagaries, and some genes under-represented on the microarray, the scope of genes and functional biology affected is likely even greater than this tally represents. Other studies place the number of diurnally regulated plant transcripts up to 35% [21], [23], with some reports displaying much higher proportions [23], [28].

By our definition diurnally cycling gene transcripts have one major peak during each cycle. The peak expression was used to group transcripts into time-of-day bins. When these genes were assigned to functional categories, and the relative representation of those functional categories was plotted across the span of the day, most functions had a marked representation for a particular time of day (Figure 4). There was a tendency, however, for some functional categories to have a bimodal pattern, wherein there was a mid-morning peak at ZT4 and a secondary peak in the late afternoon or evening at ZT12 or ZT16 (Figure 4). About 6% of the functional terms were assigned as bimodal regulated and further subdivisions are apparent according to relative enrichment of the morning or afternoon peak. Together with the functions assigned as peaking at just one time point in the day, 87.6% of the 1737 functions were assigned to one of these patterns, with just 80 assigned to “Other” and 135 to “Broad” patterns. That most of the functional categories were assigned to one of these temporal peak patterns indicates a fairly defined progression of functions across the day. Thus, functional groups are not spread uniformly across the different phases of the day, but instead often exhibit distinct patterns of representation.

**Figure 5:**
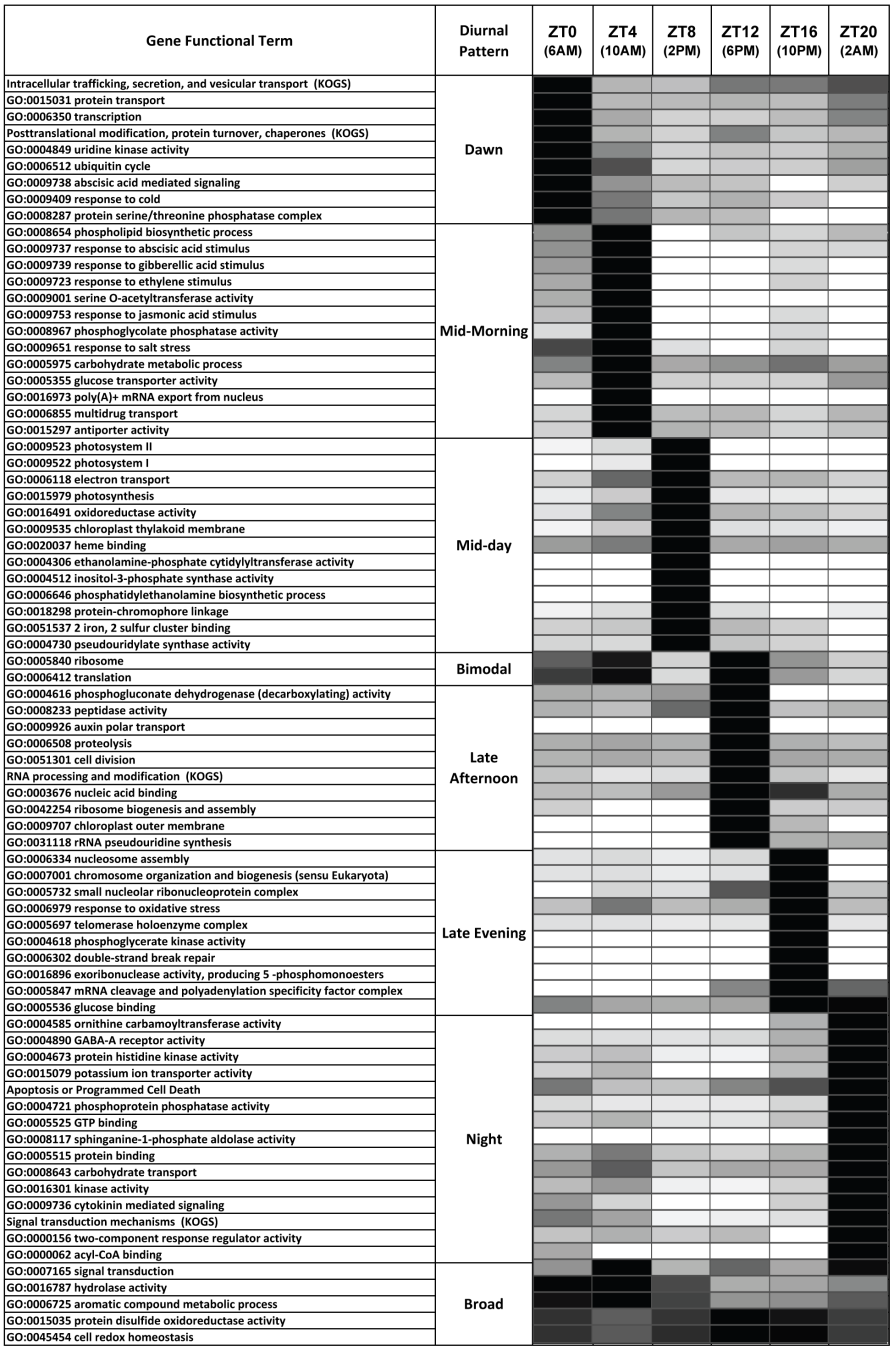
Diurnal Patterns for Temporally Enriched Gene Functional Terms. A selection of 77 of 168 functional terms that had a false discovery rate of 1% for temporal functional enrichment at particular time points in the day is presented. The gray-scale plot is the percent of maximum FEI (for the six time points). FEI stands for Functional Enrichment Index, which is the absolute Log of the binomial probability (see Materials and Methods).

In addition to this representational survey, we also subjected the functional categories to a statistical test for functional enrichment using the binomial probability test and applied cutoffs at false discovery rates of 10% and 1%, yielding 581 and 168 functional categories, respectively. At both these cutoffs the pattern of temporal enrichment across the day persisted, with functional groups showing enrichment at all six time points and falling into the same patterns. The entire list of functional categories is available in Table S4, but we have highlighted a subset of 77 that are functionally enriched (1% FDR cutoff) for attention in Figure 5.

The 1737 gene functions and their various temporal enrichments present a complex story beyond the scope of this paper to describe fully. We take this opportunity, however, to point out some prominent features of the functional enrichment across the day cycle. The dawn-enriched functional classes include processes involved in protein transport, secretion, intracellular trafficking and modifications, including ubiquitin cycle activity and the serine-threonine phosphatase complex (Figure 5). This suggests that at dawn many existing proteins are modified or trafficked within the cell to adapt to the coming day. Transcription is also enriched, indicating that new gene expression is getting underway. Interestingly, the response to cold function is also enriched in early morning; in Arabidopsis, some diurnally regulated cold response genes that peak in the morning [49] presumably relate to cooler temperatures of the night and early morning. In addition, abscisic acid mediated signaling is enriched, foreshadowing the response to abscisic acid stimulus at mid-morning ZT4.

The mid-morning (ZT4) functional enrichments are notably characterized by enrichment for multiple plant hormone response to stimulus processes, all at 1% FDR cutoff: response to stimuli from abscisic acid, gibberellic acid, ethylene, and jasmonic acid. The response to auxin stimulus peaked at ZT4 also, but exhibited a somewhat bimodal pattern, including ZT12 (6PM) enrichment. In contrast, response to cytokinin stimulus peaked at late afternoon ZT12 (6PM), and cytokinin mediated signaling peaked at night ZT20 (2AM). Cytokinin sensing was also observed to peak at night in Arabidopsis [22], and plant hormone response to stimuli were reported in the morning [50], suggesting conservation of these functional enrichments across species. In addition, the ZT4 time point includes a response to salt stress enrichment, perhaps related to that observed in Arabidopsis, which might relate to water stress issues emerging during the day [22]. The morning exhibits enrichment for a variety of small molecule transporters. Notably, sugar metabolism is well-represented by functional enrichment for carbohydrate metabolic process, glucose transporter activity, sucrose-phosphate synthase activity, inositol biosynthetic process, trehalose catabolic process, alpha-amylase activity, and starch synthase activity.

As the morning transitions to mid-day, functional enrichment becomes dominated by photosynthesis and related processes, such as photosystem I, photosystem II, and chloroplast thylakoid membrane, the glutamyl-tRNA reductase activity which is involved in heme porphyrin biosynthesis of chlorophyll [51]. Other functions are also enriched, such as membrane maintenance represented by phosphatidylethanolamine biosynthetic process, and sugar metabolism represented by glucose 1-phosphate metabolic process (Figure 5 and Table S4).

The bimodal patterned set contains two functional categories at the 1% FDR, namely GO:0005840 ribosome and GO:0006412 translation, suggesting that before and after the photosynthetic maximum at mid-day, there is active translation, presumably to prepare proteins required for photosynthesis, and then again in the afternoon to transition to a post-photosynthetic state. These two categories were about equally enriched before and after mid-day. Other interesting functions appearing at a 10% FDR, and having a bimodal pattern, but more enriched in the morning than the afternoon are: response to auxin stimulus, histone acetylation, and citrate transporter activity. Bimodal patterns with an apparent afternoon enrichment are: nitrate reductase (NADH) activity, aldehyde metabolic process, and red or far red light signaling pathway. The bimodal pattern of functional enrichment involves functional peaks typically at mid-morning (ZT4) and again later in the afternoon (ZT12) or evening (ZT16). In this experiment sunrise was 6:01–6:03 am (ZT0) and sunset at 8:41–8:39 pm (ZT14:40). Sunrise was thus 4 hours before the ZT4 functional peak, but sunset is 2.45 hours after the ZT12 time point and 1.25 hours before the ZT16 time point. Additional time points would have provided greater resolution, but it appears that the bimodal patterns are split between those that have ZT4:ZT12 and ZT4:ZT16 bimodal patterns, which may relate to this asymmetrical placement of the time points relative to the sunrise and sunset in this field-grown experiment.

By late afternoon (ZT12 or 6PM), the functional enrichment trends toward protein turnover (peptidase activity and proteolysis), and RNA metabolism (RNA processing and modification, ribosome biogenesis and assembly and rRNA pseudouridine synthesis). This presumably relates to a reworking of the cellular metabolism as photosynthesis decreases. In addition, cell division and auxin polar transport are enriched, signifying renewed growth and development. In addition there is enrichment for chromosome maintenance and repair: nuclear heterochromatin, single-stranded DNA binding, damaged DNA binding, 3′-5′ exonuclease activity, and telomere maintenance via telomerase. These themes continue into the late evening (ZT16) with enrichment for telomerase holoenzyme complex, double-strand break repair, and chromosome organization and biogenesis. In Arabidopsis cell and chromosome division and DNA and RNA synthesis were also observed to peak towards the end of the day [22]. In late evening nitrate assimilation and some amino acid biosynthesis and transport processes are enriched: nitrate assimilation, arginine biosynthetic process, L-serine biosynthetic process, and amino acid permease activity (Figure 5 and Table S4). Although nitrate assimilation was reported to peak at the end of the night in Arabidopsis, both maize (Table S4) and Arabidopsis revealed sulfate assimilation peaking at dawn or the end of night [22].

At night (ZT20) the amino acid biosynthesis enrichment theme persists in functions such as ornithine carbamoyltransferase activity, racemase and epimerase activity acting on amino acids and derivatives, tyrosine metabolic process, and tryptophan biosynthetic process. A variety of signaling processes is also enriched, including protein histidine kinase activity, two-component response regulator activity, and GABA-A receptor activity. Other gene functions show irregular patterns not obviously related to the linear progression of the day or flanking the photosynthetic maximum. In addition, many functions show a broad distribution across the day, often reflecting large functional classes with many genes such as hydrolase activity and signal transduction.

A special note on sucrose metabolism is warranted because it has been demonstrated in Arabidopsis leaves that sugars, in particular sucrose, heavily influence many diurnally regulated genes. Sucrose levels rise during the day, with starch accumulation following, and then starch depleting during the night such that it is nearly gone by dawn [22]. Functional terms bearing starch and sucrose were identified in maize (Table S4). The starch catabolic process peaked at the end of night and highest at dawn, with a similar pattern for beta-amylase activity. By mid-morning (ZT4) sucrose-phosphate synthase activity, sucrose metabolic process, and starch biosynthetic process peaked. Glucose 1-phosphate metabolic process peaked during mid-day, with response to sucrose stimulus showing a bimodal pattern. By evening sucrose transporter activity peaked. These maize findings thus appear consonant with the pattern observed in Arabidopsis that starch is catabolized at night, resynthesized in the light, accompanied by sucrose metabolism bracketing peak photosynthetic activity.

Notably, despite the great variety of genes and functions being diurnally regulated, most functional categories have only a minority of their total gene set members that are diurnally regulated. Among the 1737 functional categories, the mean set coverage was 28.2% with the median 20% and mode about 15% (Figure S5). No functional categories containing multiple genes were found to be completely covered by diurnally regulated transcripts, and few functional categories were outstandingly enriched for diurnally regulated transcripts. However, of possible significance, phosphoglucomutase activity had five of six, and auxin polar transport had three of four transcripts among the diurnal set, but these and related correlations are tentative until gene model redundancy and paralogy are more fully understood. Because the majority of genes within most functional categories are not diurnally regulated, this implies that some categories may not show a marked temporal enrichment in the day, and even if they do, individual genes within those categories could still perform decisive roles at other times in the day. We also recognize that in addition to mRNA expression assayed here, there are likely many other metabolite and protein signaling diurnal activities.

### Developing Ear Output Pathways

Despite the greatly reduced diurnal rhythms in immature ears, concentrated in the core clock components, a few apparent output genes were nonetheless found to be cycling (Table S3). The list of robust cycling transcripts includes up to 13 maize light-harvesting *CAB* transcripts (chlorophyll a-b binding protein), which is a subset of the greater maize *CAB* gene family. The *CONSTANS-like (ZmCO-like)* gene, mapped to chromosome 1, cycles in ears and leaves with a peak of expression at early evening (ZT12); however, it is a different *CO* homolog that was indentified previously as *conz1* on chromosome 9 [35]. Well-defined cycling was also detected for the MYB-like transcription factor (*ZmMyb.L*) which peaked at dawn (ZT0). This gene is a putative candidate for the maize ortholog to *REVEILLE1*, a Myb-like transcription factor integrating the circadian clock and the auxin pathway in Arabidopsis [19]. The ZmMyb.L protein has a SHAQKFF motif which is a distinctive domain of morning phase Myb-like transcription factors (Figure S6). Two ear-specific genes have intriguing functional annotations, a zinc finger protein (ZmZF-5) peaking at ZT4, and an osmotic stress/abscisic acid-activated serine/threonine protein kinase (ZmSAPK9) peaking at ZT12. Among other cycling genes there are three encoding transporters, two heat shock proteins, several enzymes, and several hypothetical proteins (Table S3).

Microarray analysis of Arabidopsis root and shoot tissue has shown that a simplified version of the core oscillator does cycle in root tissue [52]. The data from this Arabidopsis experiment was downloaded from the NASC microarray database (Accession NASCARRAYS = 493), and the same GeneTS algorithm was applied. Applying the same methodology revealed that using a 10% FDR rate, only 730 probes cycled in shoots, while no probe was statistically significant in the root tissue. Using a very permissive 50% FDR, 6518 transcripts were identified as cycling in shoot tissue compared with 335 in the root tissue (Table S5). This approximately 95% reduction in the number of cycling genes shows that while clock components, namely the morning loop, may be functioning in roots, diurnal/circadian rhythms exert little control over the global root transcriptional program. In two independent samples/species of non-light perceiving tissue, neither tissue display remarkable rhythmicity. This differs from whole-plant grown in dark, for example, where the majority of the RNA is still arising from photosynthetic tissues. Whatever signals from the leaves that animate diurnal behavior in the developing ear, their result is not transcriptional rhythmicity in the ears. At present, we are inclined to believe that source tissue does not drive diurnal expression in the sink tissue, thus the maize core oscillator would be organ-autonomous. While it is possible that non-photosynthetic tissue behavior is regulated post-transcription, this scenario seems unlikely given the key component of transcription in all current model systems.

## Discussion

### Diurnal rhythms widely regulate the leaf transcriptome

This study demonstrates that the diurnal transcriptional behavior of maize is robust and similar to that of the model plant Arabidopsis. Results from light receiving photosynthetic leaf tissue identified diurnal rhythms for 22.7% (10K/44K probes) of the expressed transcripts using the Agilent microarray technology. Increasing the density of time points might increase the number of cycling transcripts, but the overall proportion of transcripts detected as cycling in maize leaf tissue is comparable to that observed in studies with Arabidopsis [23], [25].

### Maize ear core oscillator is intact but decoupled from output pathways

In the non-photosynthetic developing ear, diurnal rhythms contributed little to the transcriptional program. Only 47 genes were identified as cycling diurnally in ears and their amplitudes were attenuated severely compared with leaves. Importantly, 11 orthologs of the core oscillator system appear in both the ear and leaf diurnally regulated gene sets. Therefore it appears that the core oscillator is active in ears in addition to leaves. The core oscillator of plants has been described as an interlocking three or four loop process [2], [53]. Our results indicate that the central feedback loop, consisting of *ZmCCA1/ZmLHY* and *ZmTOC1a/ZmTOC1b*, appears to be conserved in maize and probably serves as the main driver for transcriptional output in leaf tissue. In ear tissue, *ZmCCA1/ZmLHY* and *ZmTOC1a/ZmTOC1b* amplitudes are attenuated, reduced 83% and 94% respectively. The ear tissue cycling pattern strongly points to persistent diurnal rhythms but at decreased amplitude. If the *ZmCCA1/ZmTOC1* loop functions as the pacemaker (time synchronizer), with this attenuated amplitude, its relative contribution to signaling diurnal output genes should also be severely reduced in ears. The two exterior loops, containing such genes as *ZmPRR73/ZmPRR37*, *gigz1/gigz2* and *ZmFKF1a/ZmFKF1b* show significant reductions in wave amplitude as well.

One explanation for the decoupling of the core clock machinery from the output pathways in ears might simply be attributed to the low amplitude of the clock genes which may therefore not generate enough signaling to trigger transcription of the output pathways, or does so feebly. A few output genes whose promoters might be sensitive to lower levels of the core oscillator products are activated in ears, but the overall transcriptional outputs appear to have been effectively decoupled. Among a few output genes cycling in ears are 13 *CAB* transcripts (chlorophyll A/B binding proteins), well-established markers of diurnal expression patterns in leaves. A reduction in both the number and amplitude of diurnally expressed genes was also observed in soybean seeds versus leaves, even though developing soybean seeds remain somewhat photosynthetically active from the limited light that penetrates the pods [27].

### Diurnal Physiological Functions

We studied diurnal gene expression rhythms to assess the scope of the diurnally regulated transcriptome in maize that could lead to opportunities to improve crop performance. It is generally believed that diurnal biology evolved to improve plant adaptation to their environments, and that disruptions in clock mechanisms can reduce viability [3], [6]. We speculate that components of diurnal biology, such as the core clock mechanism or the proximal signaling mechanisms emanating from it, including those governing key physiological output functions, are not necessarily optimized for all environments. Therefore, opportunity exists for improvement in crop performance via optimizing relationships between source and sink tissues such as leaves and ears.

This study reveals many aspects of the maize diurnal mechanism, which includes core clock genes, signaling and downstream effectors genes. The diurnal pattern in maize leaf gene expression is pervasive, with thousands of genes and their attendant functions cycling in diurnal rhythms. The apparent succession of physiological roles across the span of the day is intriguing (Figures 4 and 5), and suggests specifically staged control of expression, which may be a natural progression of physiological responses to both proximal and distal events intrinsic to plant adaptation to daily rhythms. It is acknowledged that finer time point resolution will yield both more diurnally regulated transcripts, and also further delineate the succession of functions peaking across the day. Nonetheless, this genome-wide diurnal profiling survey, including assignments to over 1700 functional terms, the first reported for maize, has uncovered a durable outline of the succession of functional events throughout the day. It is clear that diurnal rhythms are complex and deeply woven into the biology of the cell, and presumably it is adaptive to have coincident or coordinated expression of cellular machinery.

The presence of the bimodal functional enrichment pattern in the morning and afternoon/evening is intriguing, and likely reflects a fundamental process to a plant's daily regimen involving pre- and post-adaptation to photosynthesis. Importantly, because individual diurnally regulated genes peak once during the day, the existence of bimodal functional categories implies that different genes within those categories are peaking at different times. This suggests that either different members of the same function, such as paralogs or gene family members, or different processes within that function, such as sequential steps in a catabolic process or antagonistic steps in regulation, are peaking on either side of the mid-day photosynthesis crest. Because these peaks flank the main period of photosynthesis, this suggests that many genes are activated to prepare the plant for photosynthesis, while others are stimulated as photosynthesis winds down for the day.

The diurnal patterns are strong in leaves, but weaker in developing ears. In ears the core clock effectively cycles, but produces feeble output signals that are likely below the threshold for effective activation of output pathways. This may be sufficient to explain the relative lack of immature ear diurnal behavior. However, developing ears are also a main sink of photosynthate, fixed carbon and sugars, from source organs experiencing the diurnal rhythms. For example, it has been demonstrated that sugars such as sucrose have a potent effect on diurnally regulated genes in Arabidopsis [22]. Even if developing ears do not have a marked transcriptional diurnal system, diurnally regulated signals such as sugars received from leaves might be expected to occur, which in turn could activate diurnal transcription of ear genes. Yet, this is apparently not the case with maize. It is not clear what provides this insulation. It may also be that any would-be arriving signals from leaves are so attenuated or temporally out of phase that they become effectively arrhythmic. It has been observed in soybean that some genes are diurnally out of phase between developing seeds and leaves [27], but this could be complicated by contributions from the intrinsic photosynthetic activity within the soybean seeds themselves. Considering the times at which the few diurnally regulated ear genes peak during the day, the functional enrichment suggests signal transduction and transcription in the morning, photosynthesis in the afternoon, and core oscillator and transcriptional regulation in the evening. Direct connections of these ear events if any to maize leaf diurnal rhythms remain to be elucidated.

## Materials and Methods

### Diurnal Tissue Collection

Plants of the public inbred line B73 were grown under field conditions in Johnston, Iowa, U.S.A. to V14–V15 stages, as defined according to the appearance of the leaf collar of the uppermost leaf [54]. For Agilent microarray profiling, samples were taken starting at daybreak on day 1 (ZT0), and taken every 4 hours hence for 72 hours. At each time point, the middle section of the top leaf, excluding the midrib, was pooled together with leaves from two separate plants to create one of three biological field replicates. The top-most immature ear, 4–5 cm in length, was also collected from the same plants at the same time. All tissues were snap-frozen in liquid nitrogen. Day length was calculated according to records from U.S. Naval Observatory, Astronomical Applications Department. Sunrise was at 6:01–6:03 a.m., and sunset was at 8:41–8:39 p.m., respectively, for 23–25 July 2008. Air and soil temperatures and precipitation were also recorded (Table 3).

**Table 3:** Air and soil temperatures and precipitation.

Date	Air High °C	Air Low °C	Soil High °C	Soil Low °C	Rain, mm
July 23 2008	27.2	16.7	26.7	21.1	0.8
July 24 2008	22.7	18.3	22.8	20.5	0.2
July 25 2008	28.9	20.0	25.5	20.0	0

### Agilent Microarray Design

The Agilent “105K” microarray chip was designed for maize genomic and transcript sequences in an effort to cover most maize genes with at least one 60-mer oligonucleotide probe. A pool of about 1.4 million public and private ESTs was consulted, derived from diverse tissues and treatments. Maize leaf and immature ears were well represented among this pool, with about 22% of the ESTs coming from leaves or similar photosynthetic aerial organs, and 12% coming from immature ears. The majority of the sequences were derived from the B73 genotype, but there were other genotypes represented. A higher quality subset of 1.1 million ESTs was used to generate a transitory transcript assembly of about 66 K transcript sequences of average length 1.0 kb. At least one 60-mer oligo was designed for nearly all of these transcripts, but at times more than one oligo was designed per transcript. In an effort to identify other genes, or perhaps parts of genes, not represented in the 66 K transcript set, and to fill out the array, various BLAST analyses were conducted against the then (in fall 2006) available public B73 genomic sequences, namely B73 BACs in GenBank and the AZM5 GSS Assembly dataset produced by TIGR. The resulting array design is believed to encompass most maize genes at least once, but there are certainly genes missing, others represented more than once, and sequence errors or genotypic variation in the dataset, or oligos designed to straddling intron-exon junctions, or to chimeric junctions inherent in ESTs and their assemblies, that may deter some oligos from functioning against a B73-specific transcript sample. Subsequent efforts to estimate the number of unique genes in this microarray suggest about 45,000 genes. This “105K” format Agilent microarray consists of 103,685 oligos designed to maize genic sequences. The Agilent microarray probe designs, plus the raw expression data, are available from GEO (Series Accession GSE23918, Platform Accession GPL10837). The balance of the space on the “105K” microarray (105,073 total oligos) was reserved for technical and internal hybridization controls.

### Microarray Analysis

Total RNA was isolated from frozen ground tissue (SQ Tissue Kit, Omega Biotek), followed by polyA RNA isolation (Illustra mRNA Purification Kit, GE Biosciences) for the 108 samples. The total RNA and polyA RNA samples were visualized and quantified on Agilent's Bioanalyzer to check for degradation and to determine the concentration. Each mRNA sample was made into double-stranded DNA, amplified by an in-vitro transcription reaction, and labeled with Cy3 or Cy5 fluorescent dye, all using Agilent's Low RNA Linear Amp kit. Biological replicates were labeled alternately using Cy3 or Cy5 to guard against dye-bias. Intensity values were determined from each of the two channels using the Rosetta Resolver Ratio-Split method [55], [56] and thereafter used as single values per sample. The cRNA product was purified with Agencourt's RNAClean Kit that utilizes SPRI (Solid Phase Reversible Immobilization) paramagnetic bead-based technology. Overnight hybridizations were performed with equal amounts of labeled cRNA loaded onto a custom 2x105K Maize Oligo Microarray from Agilent Technologies (Palo Alto, CA) according to Agilent's Two-Color Microarray-Based Gene Expression Analysis protocol. Gene expression levels were measured on the Pioneer Maize 105 K Agilent Platform. After hybridization, the microarray slides were washed and scanned immediately with Agilent's G2505B DNA Microarray Scanner at two laser power settings (100% and 10%). The images were inspected visually for image artifacts, and feature intensities were extracted, filtered, and normalized with Agilent's Feature Extraction Software (v 9.5.1). The data were then normalized using quantile normalization [57], [58] and passed forward for periodicity determination. Further quality control and downstream analysis were performed using data analysis tools in Rosetta's Resolver Database and in the statistical program R [R- Development Core Team (2008). R: A language and environment for statistical computing. R Foundation for Statistical Computing, Vienna, Austria. ISBN 3-900051-07-0, URL http://www.R-project.org]. Intensities from both leaf and ear time series were examined pre-normalization for signal to noise and intensity distributions, passing on both accounts (Figure S7). Intensities at the 0.25, 0.5 and 0.75 quantiles were comparable. Probes were considered “Present” if the average of the intensity values from the replicates exceeded 50 at least once per day. Using this methodology, 44,187 probes from leaf and 38,445 from ear were retained for periodicity analysis.

### Periodicity Determination

The GeneTS algorithm, based heavily on Fisher's G-test, was performed as described previously [38], [39], [40]. Probes considered “present” as described above were assessed for diurnal periodicity. Only those genes that were found to cycle with a 24 hour period were considered significant. The q-values were generated via the Storey method [41] and used to correct for multiple measures. A conservative FDR rate was selected in order to present the best possible data, although most likely underestimating the total number of diurnally cycling genes.

### Gene Functional Analyses

The transcript assembly was searched by tBLASTx against the UniProt UniRef100 reference proteins, but then limited to only those proteins assigned to the taxonomic sub-classification Viridiplantae. The best BLAST match by Eval of 1e^−10^ or higher significance was identified, and any Gene Ontological (GO, http://www.geneontology.org/) terms assigned to the UniRef100 protein were identified. Separately, the transcript assembly was also searched by tBLAST and parsed at the same Eval 1e^−10^ criteria for the best matches among the KOGs (Eukaryotic Clusters of Orthologous Groups, http://genome.jgi-psf.org/help/kogbrowser.html) reference proteins, and through the best match, KOGs functional category assignments were assigned to the query transcript. The 10,037 diurnally cycling Agilent array probes were matched where possible to the transcript assembly, but due to some probes matching the same transcript member, 6674 unique transcript assemblies were identified. These were then arrayed into the six time windows (ZT0, ZT4, ZT8, ZT12, ZT16, ZT20) wherein the transcript exhibited its peak expression. (In 131 instances transcripts had peaks straddling two adjacent time peaks, and so they were assigned to both). The number of unique transcripts in the entire 66 K assembly, or just the 6674 diurnally regulated transcripts across all or each separate time window, were identified and assigned to each of the GO or KOGs functional terms. Using these numbers, the binomial probability statistic was used to calculate the probability that at random each GO or KOGs functional term would have the observed number of instances among the diurnally regulated datasets. The absolute Log of the binomial probability was used and referred to as the “Functional Enrichment Index” (FEI). The significance values (p-values) from the binomial probability test were then corrected for multiple measures comparisons via conversion to q-values to assess False Discovery Rates (FDR), which are presented in Table S4 [41]. Functional enrichment patterns were manually binned performed to place the 1737 GO and KOGs functional terms into 10 temporal peak patterns plus “Other” (Table S3 and S4). A sub-selection of 77 functional categories having at least FDR q-value of 0.01 or less (sic 1% FDR) was presented in Figure 5, wherein the functional enrichment is presented as a percent of the maximum time peak's FEI. This sub-selection of the 77 from among a total of 168 with a 1% FDR also favored (a) the more descriptive and specific GO terms, (b) GO terms with more than one diurnally regulated gene, and (c) non-redundant functional roles.

## Supporting Information

Figure S1 Comparison of cycling probes between tissues.
(0.68 MB TIF)

Figure S2 The exon-intron models of ZmCCA1 and ZmLHY genes. Gene models were deduced from alignment of genomic BAC sequences and cDNA (GenBank accession NM_001154010 and NM_001138057). The ZmCCA1 gene is composed of 11 exons and 10 introns, the longest introns are intron 2 (∼9 kb) and intron 6 (∼15.6 kb). The translation start codon ATG is located in the exon 5. Untranslated 5′ UTR is divided into 5 small exons, ranging in the sizes of 40–200 bp. The ZmLHY gene is composed of 10 exons separated by 9 introns. (One of the smaller exons is apparently not covered by available ESTs). The translation start codon ATG is located in exon 5. Intron 2 is ∼30.0 kb, and intron 6 is ∼20.1 kb, among the largest introns in the maize genome.
(0.19 MB TIF)

Figure S3 Domain alignment of maize and Arabidopsis ZTL-like proteins. LOV/PAS and F-box domains are framed. Six kelch repeats are demarcated by vertical lines.
(1.85 MB TIF)

Figure S4 Validation of diurnal expression for the core oscillators ZmCCA1, ZmLHY, ZmTOC1A and ZmTOC1B by qRT-PCR. qRT-PCR was performed on RNA samples that were used for the Agilent experiments according methods in Applied Biosystems' User Bulletin number 2 at http://www3.appliedbiosystems.com/cms/groups/mcb_support/documents/generaldocuments/cms_040980.pdf.
(1.11 MB TIF)

Figure S5 Diurnally regulated genes frequency among functional terms. Distribution plot of 1738 functional terms. The y-axis, for each functional term, shows the number of transcripts found to be diurnally regulated. The x-axis, for each functional term, shows the fraction of all transcripts in the assembly that are diurnally regulated.
(0.23 MB TIF)

Figure S6 MYB-like domain alignment of the morning phase MYB-like transcription factors. Box area is a distinctive SHAQKFF motif.
(0.61 MB TIF)

Figure S7 Intensity metrics from microarray hybridizations. A) Box plots of all raw (pre-normalized) microarray hybridizations for both ear and leaf tissues. B) Histogram of intensity distributions across all arrays of pre-normalized microarray intensities.
(33.21 MB TIF)

Table S1 Summary of all mRNA profiling results.
(3.26 MB XLSX)

Table S2 A list of the 60-mer oligonucleotide sequences found to be diurnally cycling in ears or leaves.
(0.02 MB XLSX)

Table S3 A list of 47 putative genes diurnally expressed in ears.
(0.04 MB XLS)

Table S4 Leaf diurnal transcripts matching 1737 functional terms and their temporal enrichment calculations.
(0.61 MB XLSX)

Table S5 GeneTS algorithm analysis applied to Arabidopsis root and shoot RNA profiling downloaded from the NASC microarray database (Accession NASCARRAYS  = 493).
(1.43 MB XLSX)
