# Supplementary figures and images for: Maize Global Transcriptomics Reveals Pervasive Leaf Diurnal Rhythms but Rhythms in Developing Ears Are Largely Limited to the Core Oscillator
Source: PLoS One. 2010 Sep 23;5(9):e12887. doi: 10.1371/journal.pone.0012887 (PMC2944807; doi:10.1371/journal.pone.0012887)

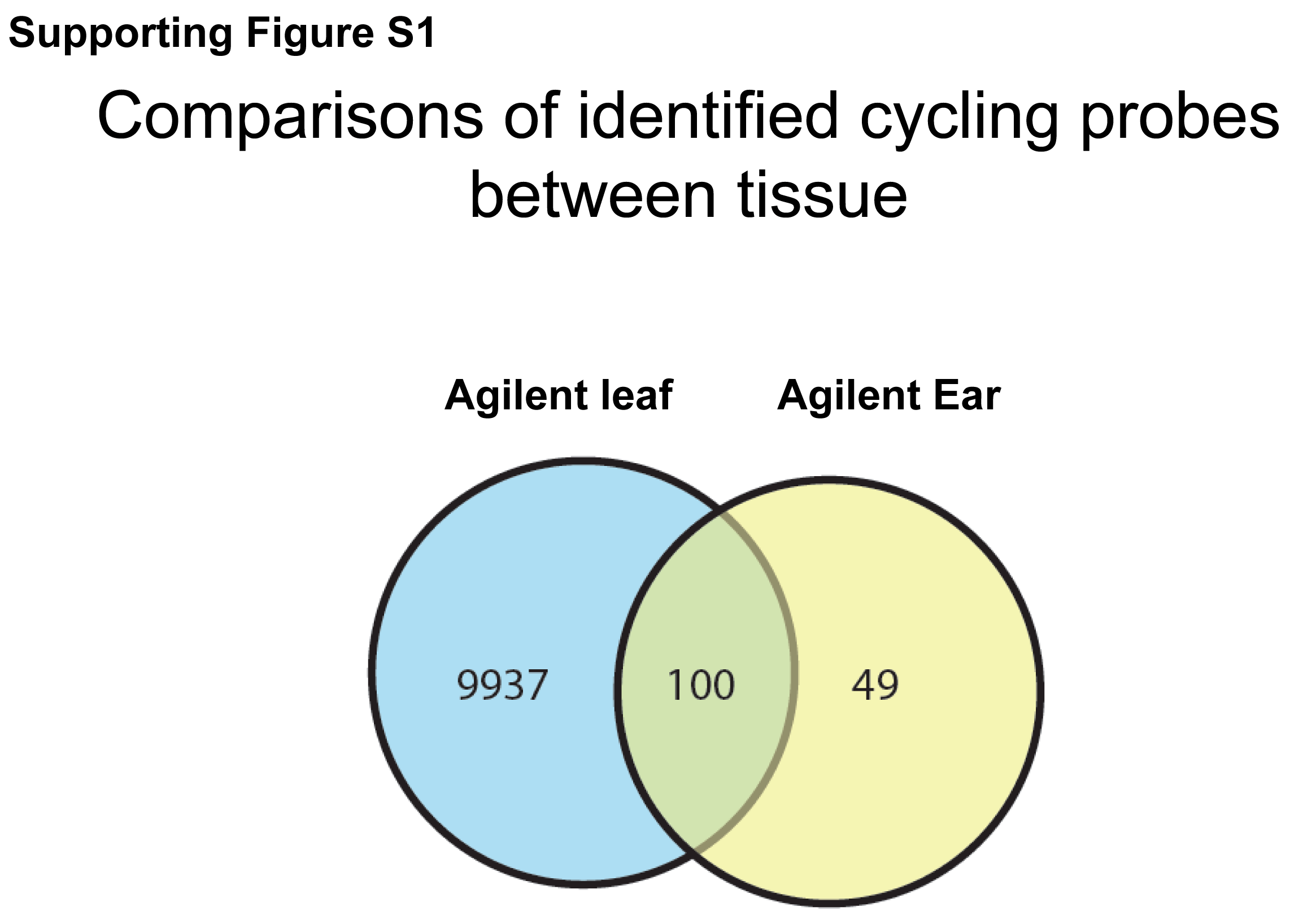

Supplement: Figure S1 — Comparison of cycling probes between tissues. (0.68 MB TIF) [file pone.0012887.s001.tif]

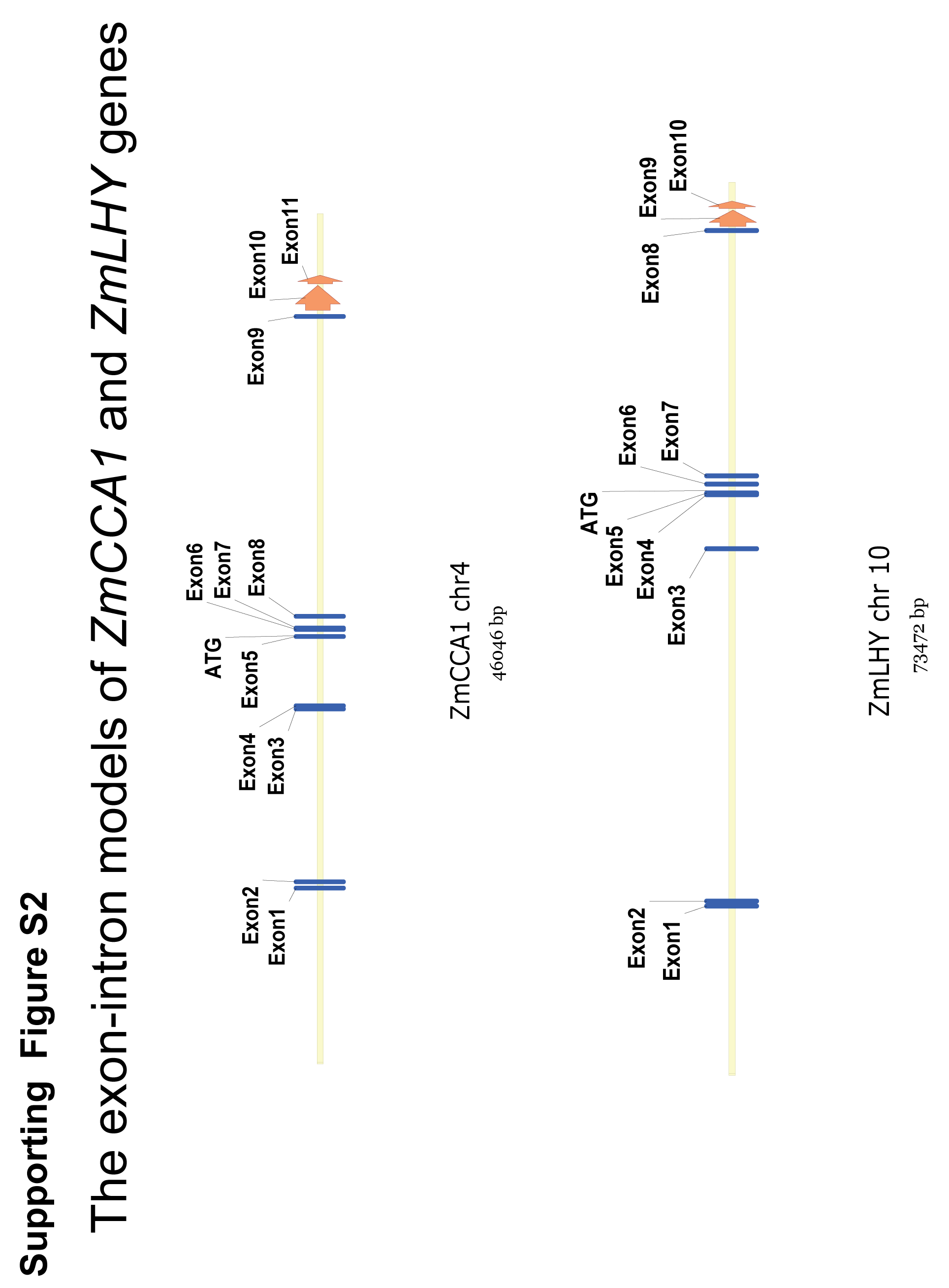

Supplement: Figure S2 — The exon-intron models of ZmCCA1 and ZmLHY genes. Gene models were deduced from alignment of genomic BAC sequences and cDNA (GenBank accession NM_001154010 and NM_001138057). The ZmCCA1 gene is composed of 11 exons and 10 introns, the longest introns are intron 2 (∼9 kb) and intron 6 (∼15.6 kb). The translation start codon ATG is located in the exon 5. Untranslated 5′ UTR is divided into 5 small exons, ranging in the sizes of 40–200 bp. The ZmLHY gene is composed of 10 exons separated by 9 introns. (One of the smaller exons is apparently not covered by available ESTs). The translation start codon ATG is located in exon 5. Intron 2 is ∼30.0 kb, and intron 6 is ∼20.1 kb, among the largest introns in the maize genome. (0.19 MB TIF) [file pone.0012887.s002.tif]

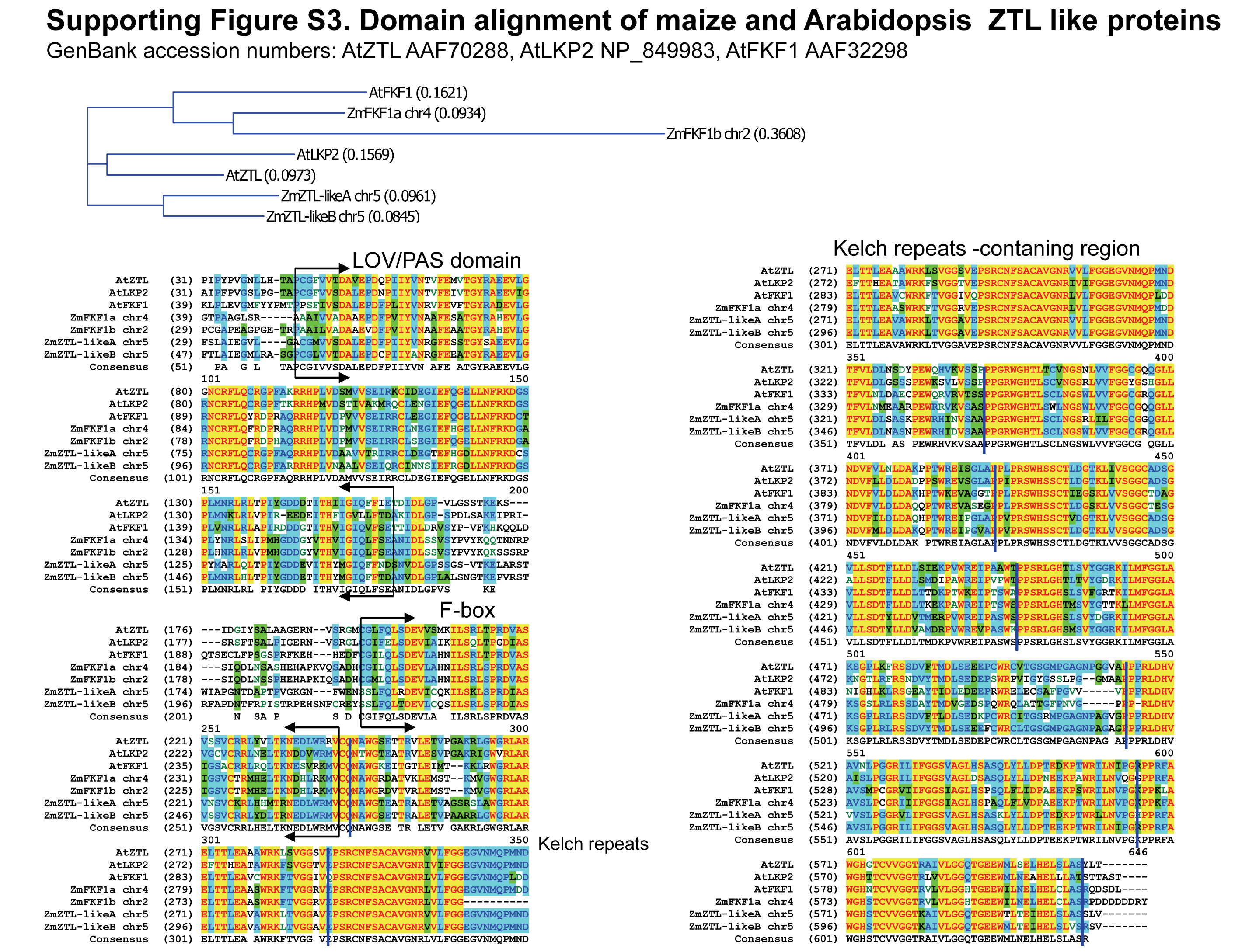

Supplement: Figure S3 — Domain alignment of maize and Arabidopsis ZTL-like proteins. LOV/PAS and F-box domains are framed. Six kelch repeats are demarcated by vertical lines. (1.85 MB TIF) [file pone.0012887.s003.tif]

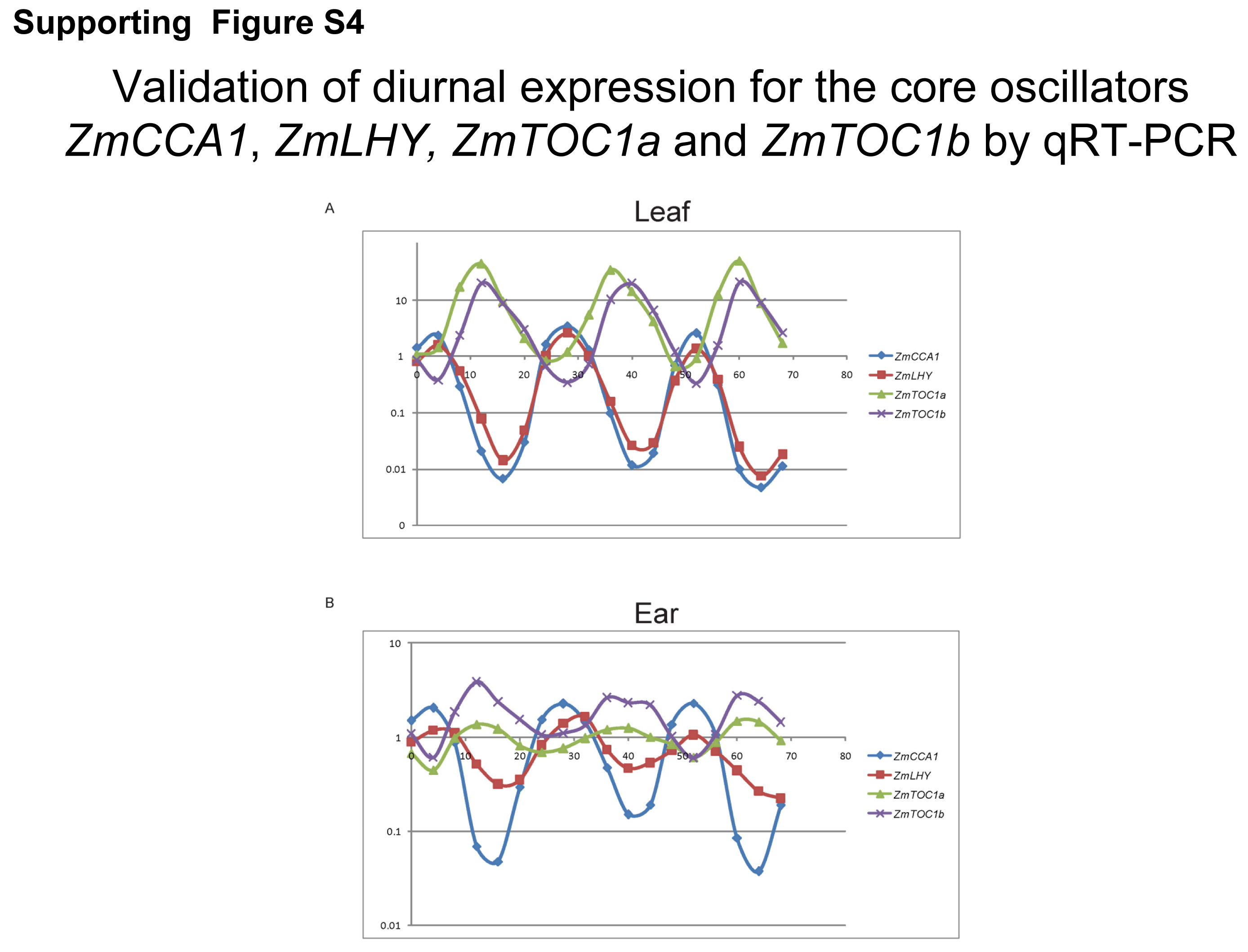

Supplement: Figure S4 — Validation of diurnal expression for the core oscillators ZmCCA1, ZmLHY, ZmTOC1A and ZmTOC1B by qRT-PCR. qRT-PCR was performed on RNA samples that were used for the Agilent experiments according methods in Applied Biosystems' User Bulletin number 2 at http://www3.appliedbiosystems.com/cms/groups/mcb_support/documents/generaldocuments/cms_040980.pdf. (1.11 MB TIF) [file pone.0012887.s004.tif]

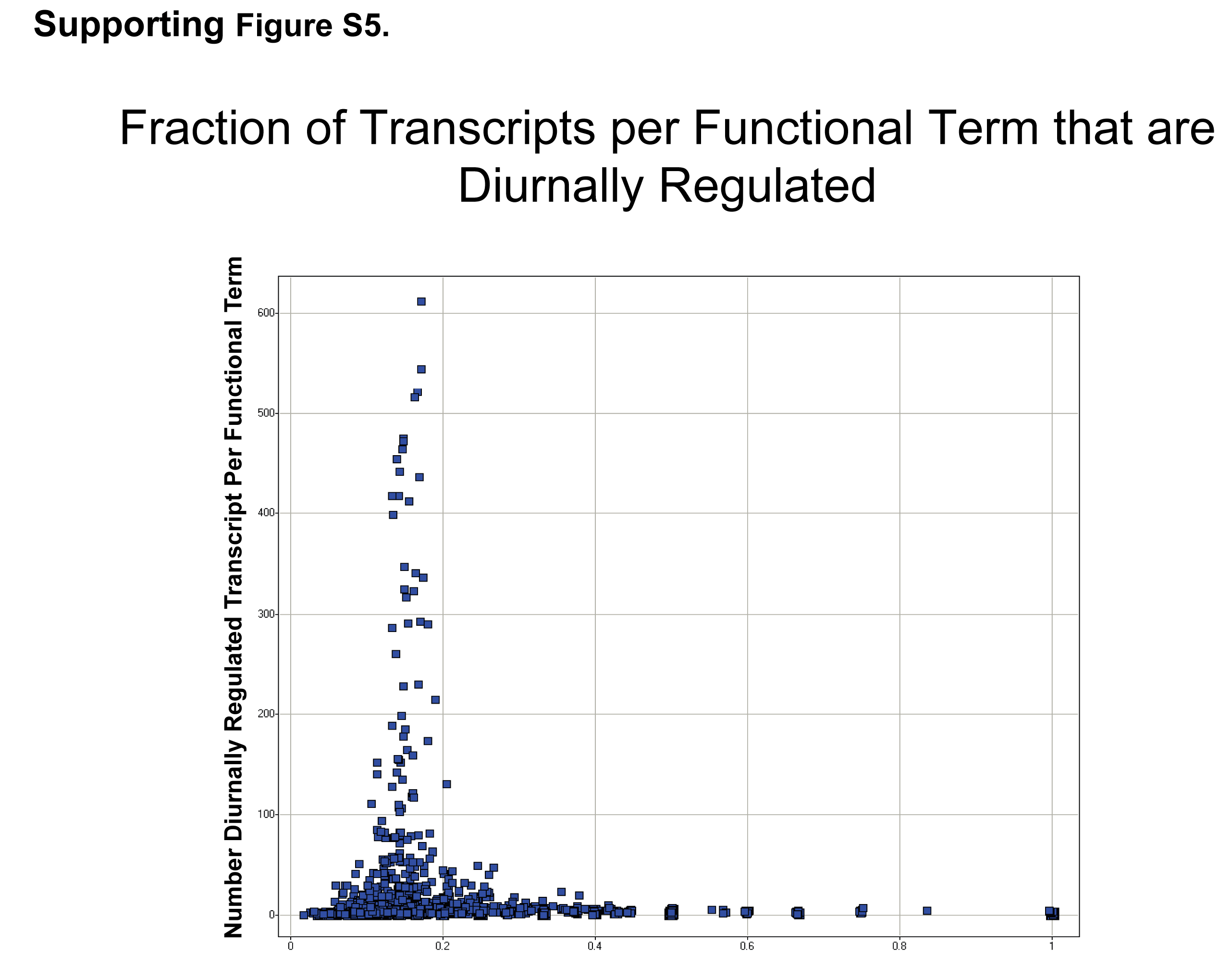

Supplement: Figure S5 — Diurnally regulated genes frequency among functional terms. Distribution plot of 1738 functional terms. The y-axis, for each functional term, shows the number of transcripts found to be diurnally regulated. The x-axis, for each functional term, shows the fraction of all transcripts in the assembly that are diurnally regulated. (0.23 MB TIF) [file pone.0012887.s005.tif]

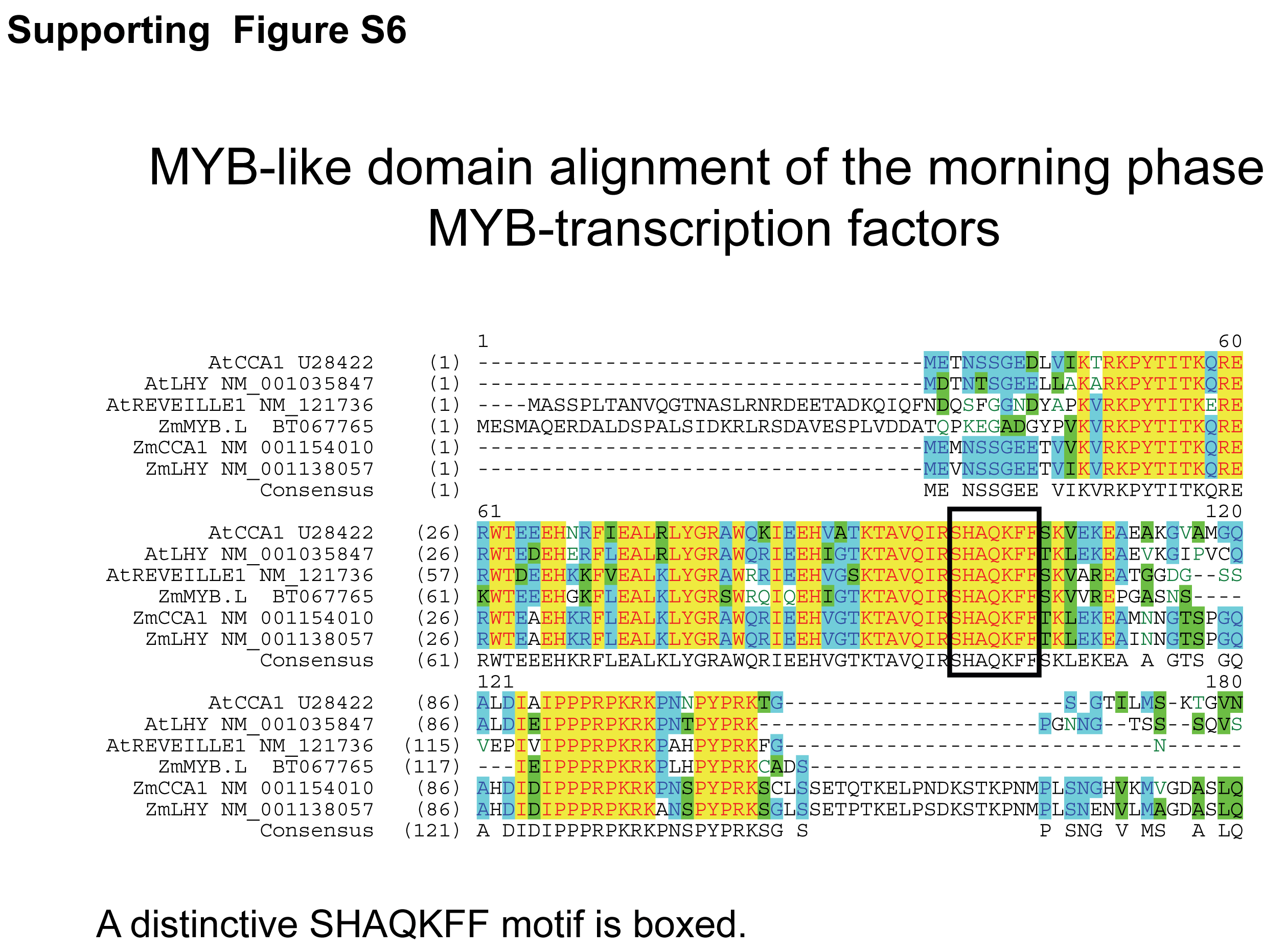

Supplement: Figure S6 — MYB-like domain alignment of the morning phase MYB-like transcription factors. Box area is a distinctive SHAQKFF motif. (0.61 MB TIF) [file pone.0012887.s006.tif]

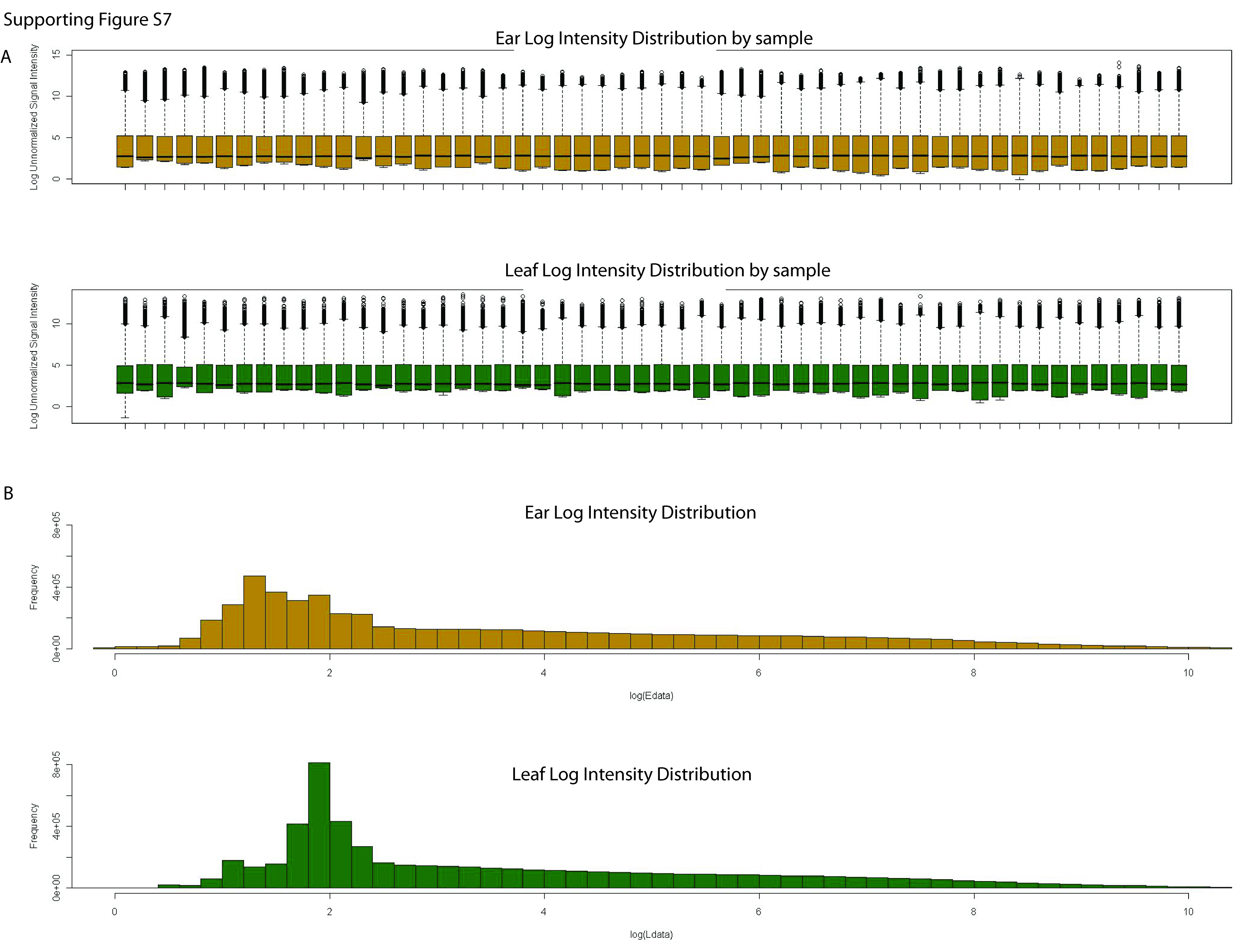

Supplement: Figure S7 — Intensity metrics from microarray hybridizations. A) Box plots of all raw (pre-normalized) microarray hybridizations for both ear and leaf tissues. B) Histogram of intensity distributions across all arrays of pre-normalized microarray intensities. (33.21 MB TIF) [file pone.0012887.s007.tif]
